# Conservative management of 11 weeks old cervical ectopic pregnancy with transvaginal ultrasound-guided combined methotrexate injection: Case Report and Literature Review

**DOI:** 10.1016/j.ijscr.2020.01.020

**Published:** 2020-01-27

**Authors:** Ipek Betul Ozcivit, Ismail Cepni, Kubra Hamzaoglu, Hakan Erenel, Rıza Madazlı

**Affiliations:** Department of Obstetrics and Gynecology, Cerrahpasa Medical Faculty, Istanbul University Cerrahpasa, Istanbul, Turkey

**Keywords:** Cervical ectopic pregnancy, Transvaginal ultrasound guided aspiration, Combined methotrexate

## Abstract

**Introduction:**

The ultimate treatment approach for cervical ectopic pregnancy remains controversial. Gestational age, serum β-hCG levels, fetal cardiac activity presence and the patient’s claim for fertility preservation are the major challenges for method of choice in each individual case. Medical treatment may be a favorable option for the treatment of late diagnosed cases, as well as the early ones.

**Presentation of case:**

In this case report, we aim to present a case of 10 4/7 weeks of cervical ectopic pregnancy successfully treated with transvaginal ultrasound-guided local and systemic methotrexate injection.

**Discussion:**

The case presented here is exceptional because even though the advanced gestational age, presence of fetal cardiac activity and high serum β-hCG values, the abortion has occurred successfully. Conservative treatment without any need of further surgical intervention was sufficient for full recovery with the preservation of reproductive capacity.

**Conclusion:**

In conclusion, transvaginal ultrasound guided local and systemic methotrexate injection may be performed successfully for the cases of advanced gestational age with fetal cardiac activity and high serum β-hCG levels.

## Highlights

• Methorexate injection in advanced gestational aged cervical pregnancy.
• Intraamniotic and systemic methorexate injection guided by transvaginal ultrasound.
• Approach to cervical pregnancy with fetal cardiac activity and high serum β-hCG levels.

## 1 Introduction

Ectopic pregnancy, defined as the implantation of the blastocyst outside the uterine cavity, occurs in 1.9% of the reported pregnancies [1]. Cervical pregnancy which is the rarest form of ectopic pregnancies, has an incidence varying from 1/1000 to 1/50,000 and is associated with high morbidity and mortality [2]. Several risk factors are associated with cervical pregnancy such as the use of intrauterine devices, history of abortions, local endometrial injury (prior cesarean section, uterine curettage etc.) and IVF treatment with embryo transfer. The most common presenting symptom of the cervical pregnancy is painless vaginal bleeding. Patients suffering cervical ectopic pregnancy, have a high risk of severe hemorrhage that may require hysterectomy, therefore timely intervention is crucial [3].

There has been several treatment approaches of cervical ectopic pregnancy. The classical treatment modality is hysterectomy which is an unfavorable approach in terms of fertility preservation. On the other hand, medical treatment may be an option for the stable patients whose diagnosis is made early. Currently, improved access to transvaginal ultrasound scanning facilities and the rapid assay of serum hCG make early diagnosis possible. Chemotherapeutic agent methotrexate, and prostaglandin analogue mifepristone and misoprostol have all been used successfully to terminate cervical ectopic pregnancy medically, usually in combination with interventional measures. There has been several cases in literature, where ultrasound-guided injection of methotrexate or potassium chloride (KCl) directly into the gestational sac or fetus has been performed. In this report which is prepared in line with SCARE criteria [14], we present a case of 11 weeks old cervical ectopic pregnancy with fetal heart activity for discussion of the conservative management success.

## 2 Presentation of case

**Fig. 1:**
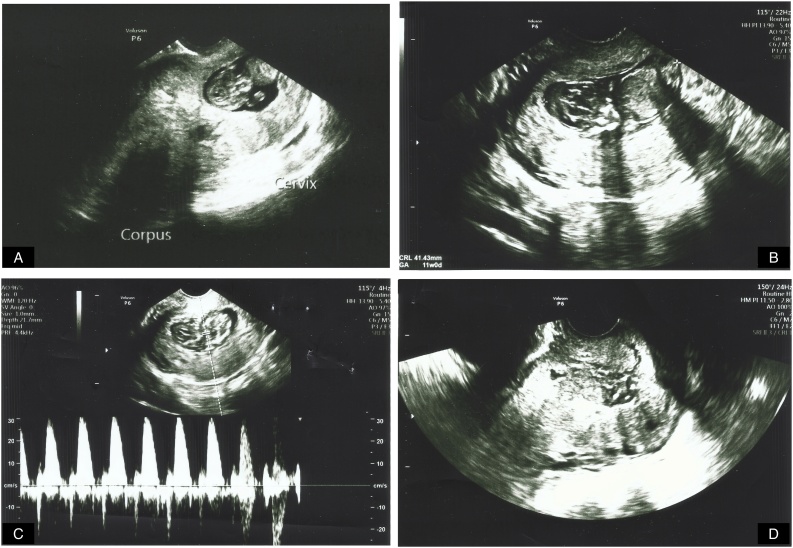
Cervical ectopic pregnancy images on transvaginal ultrasound. A. Empty uterine cavity, embryo in cervical canal. B. 11 weeks old embryo (CRL: 41.43 mm) in cervical canal. C. Embryo with fetal cardiac activity. D. Cervical ectopic locus after β-hCG levels became negative.

A 37 year-old nulliparous woman was referred to our gynecology department with a diagnosis of cervical ectopic pregnancy. According to her last menstrual period, she was 10 weeks 4 days pregnant (concordant with ultrasound). In transvaginal ultrasonography examination, embryo with crown-rump length (CRL) 41.43 mm (11 w) and a fetal cardiac activity was observed in cervical region (Fig. 1A, B and C). Her past medical and gynecologic history was unremarkable. Her vital signs and general physical examination was normal, without any defense or rebound in abdomen. There was not any bleeding observed in her vaginal examination. Her β-hCG level was more than 10.000 mIU/mL and the hematocrit level was 36.4%.

Treatment modalities, their potential risks and the severity of the condition were discussed with the patient and her husband. Because the patient is nulliparous and wishes to preserve her fertility, conservative treatment with transvaginal ultrasound-guided local and systemic methotrexate injection was recommended by our team. On 10th week 6 days of pregnancy (by LMP), with the guidance of transvaginal ultrasound, approximately 6 cc of intraamniotic fluid was aspirated by using 16-G 40 mm aspiration needle, in the operating room with sedation. 75 mg methotrexate diluted by 10 cc of physiologic serum solution had been prepared before the procedure. 6 cc of this solution was injected intra-amniotically for the local effect and the rest was injected intramuscularly for the systemic effect. After the procedure, the fetal cardiac activity was still observed in the operating room and no active hemorrhage from the cervical cavity was observed.

**Fig. 2:**
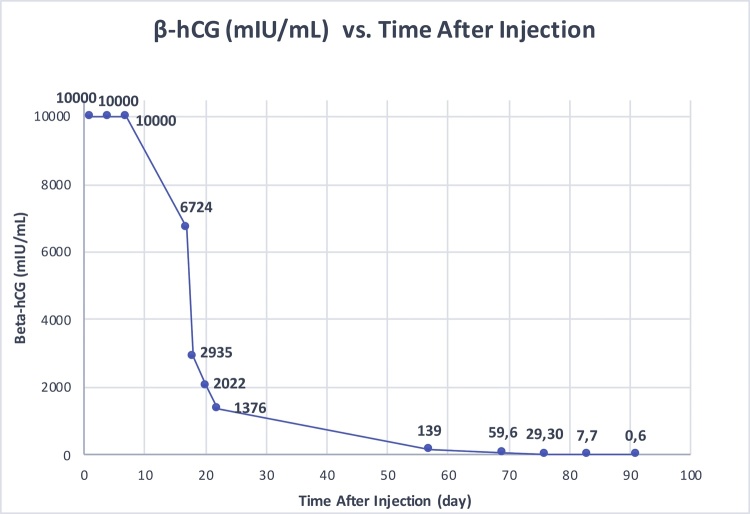
Regression pattern of β-hCG levels.

On the 3^rd^ day of methotrexate injection, fetal bradycardia was observed followed by the absence of fetal cardiac activity on 4th day. On the 7th day, the symptom-free patient, with β-hCG level above 10.000 mIU/mL and hematocrit level 33.9%, was offered discharge with the condition of regular evaluations to be conducted in the outpatient clinic. The patient preferred to stay. On the 17th day of methotrexate injection, a gestational sac of 47 × 35 mm and cervix length 25 mm was observed by transvaginal ultrasound and β-hCG level decreased to 6724 mIU/mL (Fig. 2). On the 18th and 20th day, the β-hCG levels were 2935 mIU/mL and 2022 mIU/mL, respectively, with stable hematocrit levels. On the 22nd day of the procedure, the patient complained of vaginal spotting for the first time and her β-hCG was 1376 mIU/mL. The patient discharged from the hospital on the 23^rd^ of the procedure to be followed up in outpatient clinic.

On the 57th day of the procedure, the patient admitted to our outpatient clinic without any symptoms; her β-hCG level was 139 mIU/mL; 4 × 5.4 cm cervical ectopic locus was observed by transvaginal ultrasound. Weekly β-hCG follow up recommended, till negative values of β-hCG were reached. On the 69th, 76th and 83th day of the procedure, β-hCG levels of 59.6 mIU/mL, 29.3 mIU/mL and 7.7 mIU/mL were obtained, respectively. The patient’s β-hCG value became negative (0.6 mIU/mL) on the 91th day of the injection and she had her first menstrual period next day, on the 92th day of the procedure. A week later, after her menstruation had finished, 30 × 11 mm of cervical ectopic locus was observed by transvaginal ultrasound (Fig. 1D) and spontaneous follow up was recommended.

## 3 Discussion

The advances in technology, like urinary early pregnancy test kits or easy access to ultrasound facilities, make early diagnosis of the cervical ectopic pregnancy possible while reducing the morbidity and mortality rates [4].

In 1978, before Raskin reported the ultrasonographic diagnosis of cervical ectopic pregnancy, the diagnosis remained on histopathological criteria defined by Rubin on hysterectomy specimens in 1911 [5,6]. The criteria for the cervical pregnancy diagnosis on ultrasound includes the placental implantation in the cervix, empty uterine cavity, small fundus and enlarged cervical canal, an hourglass-shaped uterus, closed internal os and unchanged sac appearance with repeated scanning with or without fetal cardiac activity. Early intrauterine pregnancy, incomplete abortion, large Nabothian cysts might be included in the differential diagnosis of cervical ectopic pregnancy [3]. Yet, for accurate diagnosis, expectant management and repeated scanning can be considered.

Since cervical pregnancy is a very rare condition, there are only case reports in the literature, but not enough studies to determine an appropriate therapeutic approach. Several treatment modalities are available: tamponade with Foley catheter, reduction of blood supply (by cervical cerclage, vaginal ligation of cervical arteries, uterine ligations, arterial embolization, surgical excision of the trophoblast, intraamniotic feticide) and systemic chemotherapy; and they should be offered to the patient in experienced special tertiary care centers [7]. Expectant management remains controversial.

The treatment for cervical ectopic pregnancy should be individualized. Mostly, the aim for preservation of reproductive potential determines the treatment modality in cervical pregnancies diagnosed early. Conservative management is the therapy of choice in a stable patient, but its success depends on gestational age and anatomy of the cervix, which is mostly formed of fibrous connective tissue, unable to respond to mechanical hemostasis and uterotonic medications [8]. Systemic methotrexate is the most common conservative treatment with a success rate of 91%. But, primary methotrexate intramuscular injection is associated with a higher failure rate when fetal cardiac activity, initial serum β-hCG level >10,000 mIU/mL, gestational age of >9 weeks and a crown-rump length of >10 mm is observed [9]. Intra-amniotic injection of potassium chloride may enhance the therapeutic effect of methotrexate. But, the recent cases mostly focus on intraamniotic methotrexate injection rather than potassium chloride or systemic methotrexate or combination of modalities [10,11].

In our facility, we had an experience on conservative management of 7 weeks old cervical ectopic pregnancy (CRL of embryo was 10 mm, consistent with a 7 week pregnancy), where we performed transvaginal ultrasound guided aspiration of gestational sac contents and systemic methotrexate injection [3]. In our current case, we dealt with a cervical pregnancy at 11^th^ week of gestation with fetal cardiac activity, CRL of 41 mm and serum β-hCG levels above 10,000 mIU/mL. Since this case had an advanced gestational age and an embryo with bigger CRL, we preferred to perform both local and systemic methotrexate injection in order to minimize our failure rate and avoid further invasive interventions. We managed to treat the cervical ectopic pregnancy successfully without any major complication or need for a second adjunctive treatment modality while preserving fertility. Because the risk of a potential hemorrhage was significant, we followed our patient 23 days in hospital and 91 days in total, until less than 5 mIU/mL of serum β-hCG levels had been reached. Since the rarity of this condition, there is not an approved cut-off point for follow up. In Kirk’s study where 7 cervical ectopic pregnancies treated with different conservative modalities, the mean observation time in hospital was 19 days, whereas the total observation time varies [12]. In our previous case, follow-up for 42 days was enough for obtaining negative β-hCG levels. But during this period the patient needed 2 U of whole blood transfusion due to heavy vaginal bleeding which was resolved after the evacuation of trophoblastic tissue with winter forceps. [3]. While performing the local injection, it was important aspirate and inject approximately equal amounts of fluid into the cervical cavity in order not to change the internal pressure and cause any type of bleeding. The internal pressure change of cervical cavity after the aspiration may be the reason of the heavy vaginal bleeding in our previous case [3].

Bleeding is the most common symptom and complication in cervical ectopic pregnancy, because of the scarcity of smooth muscle tissue in cervical region. Therefore, the other treatment modalities such as suction curettage, usually leads to severe bleeding and shock that may result in hysterectomy or even mortality. Arterial embolization has also proved to be a successful method for the control of massive hemorrhage [13].

Rarely, cervical pregnancies, especially cervico-isthmic and cervico-isthmic corporeal pregnancies persist to advanced gestation. Since placental abnormalities, like placenta accreata, accompany most of them; massive hemorrhage that may endanger patient’s life may be encountered. In a case report presented by Avery et al., massive hemorrhage after C-section of a term cervico-isthmic corporeal pregnancy was managed successfully by relaparotomy, massive blood and blood product transfusions and long hospitalization [15].

## 4 Conclusion

In conclusion, even though there is not a universally accepted treatment modality for cervical ectopic pregnancy, the major goal of conservative management for early diagnosed cases is full recovery of the patient without any further surgical interventions and preservation of fertility. Combination of local and systemic methotrexate injection may be performed successfully for the cases of advanced gestational age with fetal cardiac activity and high serum β-hCG levels.

## Ethical approval

Ethical approval has not been requested, because the methotrexate treatment is an accepted treatment modality in ectopic pregnancy.

## Consent

Written informed consent was obtained from the patient for publication of this case report and accompanying images. A copy of the written consent is available for review by the Editor-in-Chief of this journal on request.

## Registration of research studies

The study was designed as a case report, we didn’t register for UIN.

## Provenance and peer review

Not commissioned, externally peer-reviewed.

## Guarantor

Ismail Cepni.

## Funding

This research did not receive any specific grant from funding agencies in the public, commercial, or not-for-profit sectors.

## CRediT authorship contribution statement

**Ipek Betul Ozcivit:** Writing - original draft, Project administration. **Ismail Cepni:** Conceptualization, Methodology. **Kubra Hamzaoglu:** Investigation, Data curation. **Hakan Erenel:** Writing - review & editing. **Rıza Madazlı:** Supervision.

## Declaration of Competing Interest

None.
